# Partitioning the Right Ventricle Into 15 Segments and Decomposing Its Motion Using 3D Echocardiography-Based Models: The Updated ReVISION Method

**DOI:** 10.3389/fcvm.2021.622118

**Published:** 2021-03-04

**Authors:** Márton Tokodi, Levente Staub, Ádám Budai, Bálint Károly Lakatos, Máté Csákvári, Ferenc Imre Suhai, Liliána Szabó, Alexandra Fábián, Hajnalka Vágó, Zoltán Tősér, Béla Merkely, Attila Kovács

**Affiliations:** ^1^Heart and Vascular Center, Semmelweis University, Budapest, Hungary; ^2^Argus Cognitive, Inc., Lebanon, NH, United States; ^3^Department of Automation and Applied Informatics, Budapest University of Technology and Economics, Budapest, Hungary

**Keywords:** 3D echocardiography, right ventricle, right ventricular function, right ventricular mechanics, decomposed wall motion

## Abstract

Three main mechanisms contribute to global right ventricular (RV) function: longitudinal shortening, radial displacement of the RV free wall (bellows effect), and anteroposterior shortening (as a consequence of left ventricular contraction). Since the importance of these mechanisms may vary in different cardiac conditions, a technology being able to assess their relative influence on the global RV pump function could help to clarify the pathophysiology and the mechanical adaptation of the chamber. Previously, we have introduced our 3D echocardiography (3DE)-based solution—the Right VentrIcular Separate wall motIon quantificatiON (ReVISION) method—for the quantification of the relative contribution of the three aforementioned mechanisms to global RV ejection fraction (EF). Since then, our approach has been applied in several clinical scenarios, and its strengths have been demonstrated in the in-depth characterization of RV mechanical pattern and the prognostication of patients even in the face of maintained RV EF. Recently, various new features have been implemented in our software solution to enable the convenient, standardized, and more comprehensive analysis of RV function. Accordingly, in our current technical paper, we aim to provide a detailed description of the latest version of the ReVISION method with special regards to the volumetric partitioning of the RV and the calculation of longitudinal, circumferential, and area strains using 3DE datasets. We also report the results of the comparison between 3DE- and cardiac magnetic resonance imaging-derived RV parameters, where we found a robust agreement in our advanced 3D metrics between the two modalities. In conclusion, the ReVISION method may provide novel insights into global and also segmental RV function by defining parameters that are potentially more sensitive and predictive compared to conventional echocardiographic measurements in the context of different cardiac diseases.

## Introduction

For many years, emphasis in clinical cardiology was placed on left ventricular (LV) performance, overshadowing the study of the right ventricle (RV). However, RV function has been recently proven to be an important prognostic factor in heart failure with reduced or preserved ejection fraction (EF) and pulmonary hypertension (1–4). Moreover, the precise assessment of RV function has emerged as a cornerstone of patient management in specific subgroups, such as in patients with mechanical circulatory support devices or grown-up congenital heart disease (5–7). Therefore, the detailed evaluation of RV function to detect even subtle but prognostic changes and to support clinical decision-making represents a compelling demand.

Mechanistically, the RV shows a distinctive contractile pattern with three main mechanisms: (i) shortening along the longitudinal axis with the traction of the tricuspid annulus toward the apex; (ii) inward (radial) movement of the RV free wall (often referred to as the “bellows effect”); and (iii) bulging of the interventricular septum into the RV during the LV contraction and stretching of the free wall over the septum (causing shortening along the anteroposterior direction) (3, 8). Since the importance of these mechanisms may vary in different cardiac conditions, a technology being able to assess their relative influence on the global RV pump function could help to clarify the pathophysiology and the mechanical adaptation of the RV (3).

For this purpose, we developed the Right VentrIcular Separate wall motIon quantificatiON (ReVISION) method a few years ago, which is a 3D echocardiography (3DE)-based solution for the quantification of the relative contribution of longitudinal, radial, and anteroposterior shortening to global RV EF (9). Since then, our technology has been applied in several clinical scenarios (10–12), and the strengths of the ReVISION method have been demonstrated in the in-depth characterization of RV mechanical pattern and the prognostication of patients even in the face of maintained RV EF (13). The ReVISION method and the associated online platform (demo version available at https://www.revisionmethod.com) are improved continuously, and recently, various new features, such as the assessment of longitudinal, circumferential, and area strains have been implemented to enable the convenient, standardized, and more comprehensive analysis of RV function using 3DE datasets.

In addition to the parameters of global ventricular function and geometry, segmental metrics bear clinically relevant information. Concerning the LV, standardized segmentation is widely performed in different cardiovascular imaging modalities mainly to correlate regional dysfunction with coronary perfusion territories (14) or to appreciate and quantify distinct patterns in LV myocardial function, which could be a characteristic of certain pathological processes (15). The same applies to the RV, as pulmonary hypertension or arrhythmogenic cardiomyopathy are just two clinical examples among several others, where established regional dysfunction exists (1, 16, 17). Nevertheless, only a few options are available for the comprehensive and quantitative assessment of the regional RV function due to its complex 3D shape and mechanics. Therefore, we designed and implemented a volumetric segmentation (i.e., partitioning) algorithm in our current software solution.

In this technical paper, we aimed to provide a detailed description of the updated ReVISION analysis pipeline with special regards to the volumetric partitioning of the RV cavity and the calculation of longitudinal, circumferential, and area strains using 3DE datasets. We also sought to compare our echocardiography-based findings concerning the relative contribution of the three aforementioned motion components with those obtained by cardiac magnetic resonance imaging (CMRI)-based 3D reconstruction.

## Materials and Methods

### The ReVISION Analysis Pipeline

**Figure 1:**
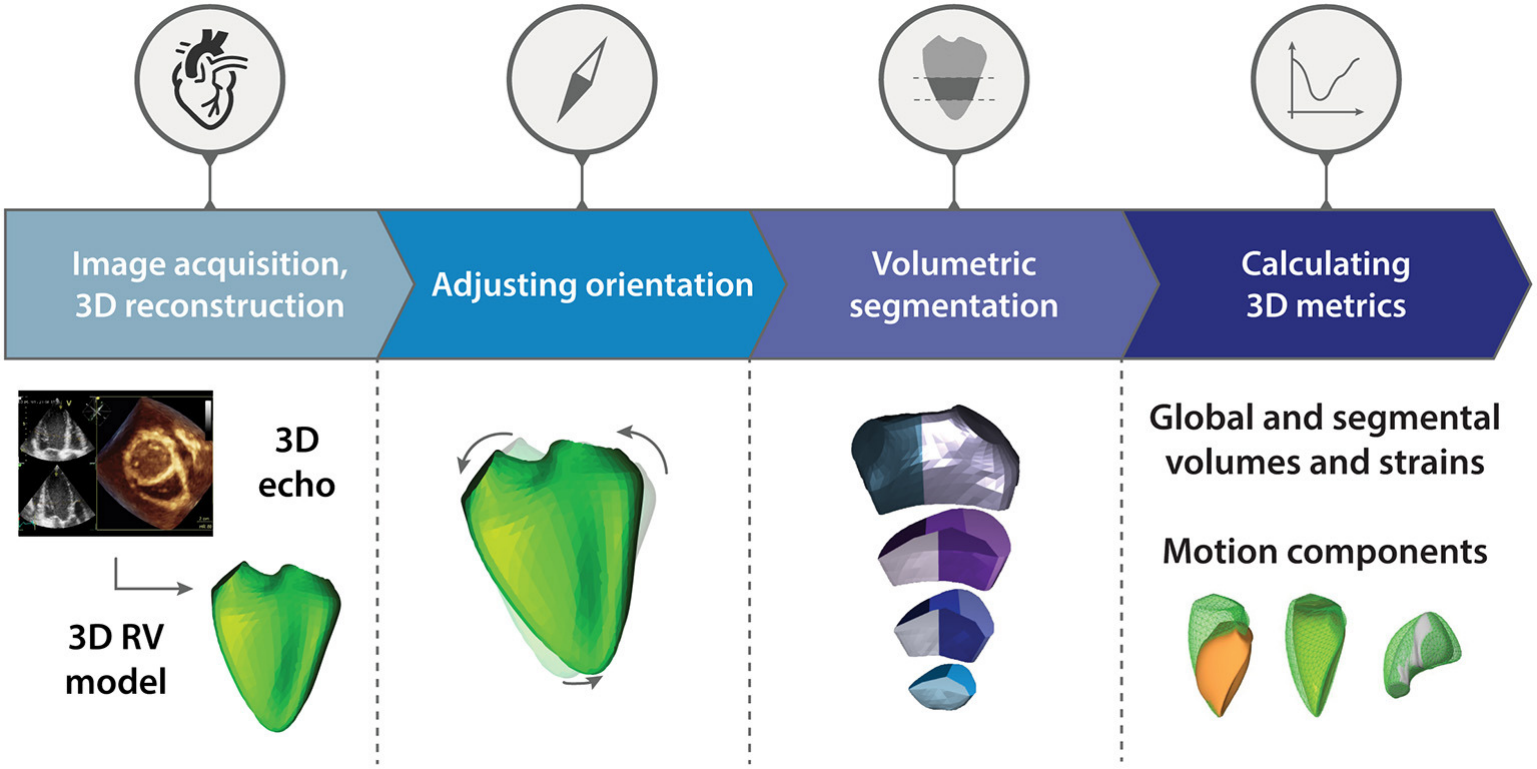
Schematic illustration of the Right VentrIcular Separate wall motIon quantificatiON (ReVISION) analysis pipeline. The updated ReVISION analysis pipeline comprises four consecutive steps: (i) image acquisition and 3D right ventricular model reconstruction, (ii) adjusting orientation, (iii) volumetric segmentation, and (iv) calculation of global and segmental 3D metrics. See text for further details. RV, right ventricle.

The updated ReVISION analysis pipeline comprises four consecutive steps: (i) image acquisition and 3D RV model reconstruction, (ii) adjusting orientation, (iii) volumetric segmentation, and (iv) calculation of global and segmental metrics, including the quantification of the relative contribution of longitudinal, radial, and anteroposterior motion components (Figure 1). Our software solution was implemented as a user-friendly and convenient online platform (https://www.revisionmethod.com), where the user can upload and analyze the reconstructed 3D models of the RV. The analytical components were written in C++, relying on the Eigen linear algebra library (version 3.3.7). The rest of the software stack uses the Play Framework (Scala, version 2.8.2) in the backend and Typescript (version 3.9.5) in the frontend.

#### Image Acquisition and 3D RV Model Reconstruction

3DE datasets can be acquired with 3D capable commercially available cardiac ultrasound systems. Then, these datasets are required to be processed using a dedicated software solution (4D RV-Function 2, TomTec Imaging, Unterschleissheim, Germany) to generate 3D models of the RV suitable for the ReVISION analysis. Following the image acquisition and 3D model reconstruction process, a series of UCD files and a header file can be exported for each subject from the dedicated software. Each UCD file contains a 3D polygon mesh representing a time instant (a frame) in the cardiac cycle. Each vertex in a mesh corresponds to a specific anatomical position, and this correspondence is consistent across time instants and patients. These files serve as the input for the next steps of the analysis.

#### Adjusting Orientation

**Figure 2:**
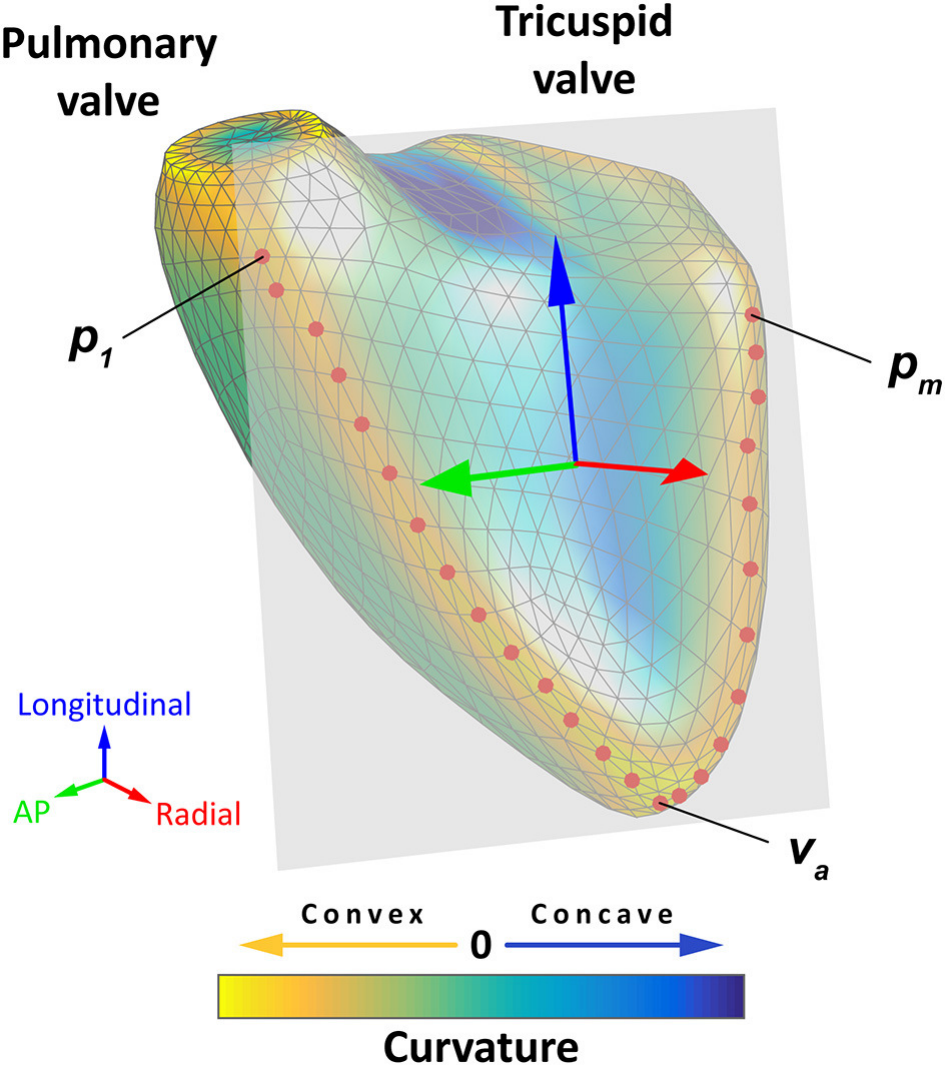
Schematic representation of the orientation adjustment. A local coordinate system is defined for each mesh series, where the basis vectors correspond to the longitudinal (blue arrow), radial (red arrow), and anteroposterior directions (green arrow). First, the points of the septum–free wall boundary (red dots) are selected automatically from predefined groups of vertices so that they have a maximal local mean curvature. Then, a plane is fitted to the selected points using orthogonal distance regression (gray plane), and the radial basis vector is defined as the normal vector of this plane. The longitudinal basis vector should point from the apex (*v*_*a*_) toward the midpoint of the most basal vertex of the anterior and the most basal vertex of the posterior septum–free wall boundary ($\frac{p_{1}+\;p_{m}}{2}$, where *p*_1_ is the most basal vertex of the anterior and *p*_*m*_ is the most basal vertex of the posterior septum–free wall boundary). These two points are projected to the plane defined by the radial basis vector, and they are used to define the longitudinal basis vector. Finally, the anteroposterior basis vector can be determined using the other two basis vectors. See text for further details. The 3D right ventricular model is visualized from anteroseptal point of view. The surface of the mesh is color-coded based on the local mean curvature: yellowish colors indicate the most convex surface, whereas the deepest blue colors correspond to the most concave surface. AP, anteroposterior.

The exported files contain a series of *m*_1_, …, *m*_*n*_ meshes where $m_{i}=\;\left\{\left(x_{1}^{i},y_{1}^{i},z_{1}^{i}\right),\;\ldots ,\left(x_{k}^{i},y_{k}^{i},z_{k}^{i}\right)\right\}$ denotes a set of 3D coordinates. A local coordinate system is defined for the end-diastolic mesh of each mesh series, where the basis vectors correspond to the longitudinal, radial, and anteroposterior directions (Figure 2). We denote these basis vectors as $B_{l},B_{r},B_{a}\in R^{3}$, respectively, and we use them to transform each mesh (*m*_*i*_) of the given mesh series into ${\hat{m}}_{i}=\;\left\{B^{-1}v_{k}|v_{k}\in m_{i}\;\right\}$ where *B* = [*B*_*l*_, *B*_*r*_, *B*_*a*_ ].

To define *B*, the following multistep analysis is performed. First, the points of the septum–free wall boundary (*P* = {*p*_1_, *p*_2_, …, *p*_*m*_}) are selected automatically from predefined groups of vertices so that they have a maximal local mean curvature. Then, a plane is fitted to the selected points using orthogonal distance regression (18), and *B*_*r*_ is defined as the normal vector of this plane. *B*_*l*_ should point from the apex (*v*_*a*_ – the vertex corresponding to the apex) toward the midpoint of the most basal vertex of the anterior and the most basal vertex of the posterior septum–free wall boundary ($v_{e}=\;\frac{p_{1}+\;p_{m}}{2}$, where *p*_1_ is the most basal vertex of the anterior and *p*_*m*_ is the most basal vertex of the posterior septum–free wall boundary). These two points are projected to the plane defined by *B*_*r*_ and denoted as ê and â, respectively. Then, the longitudinal basis vector is defined as $B_{l}=\;\frac{ê-â}{\left||ê-â|\right|}$. Finally, we define the anteroposterior basis vector as *B*_*a*_ = *B*_*r*_ × *B*_*l*_. As the result of the orientation adjustment, the longitudinal direction will correspond to the vertical axis, and the radial and anteroposterior directions will be parallel to the horizontal plane.

#### Volumetric Segmentation

**Figure 3:**
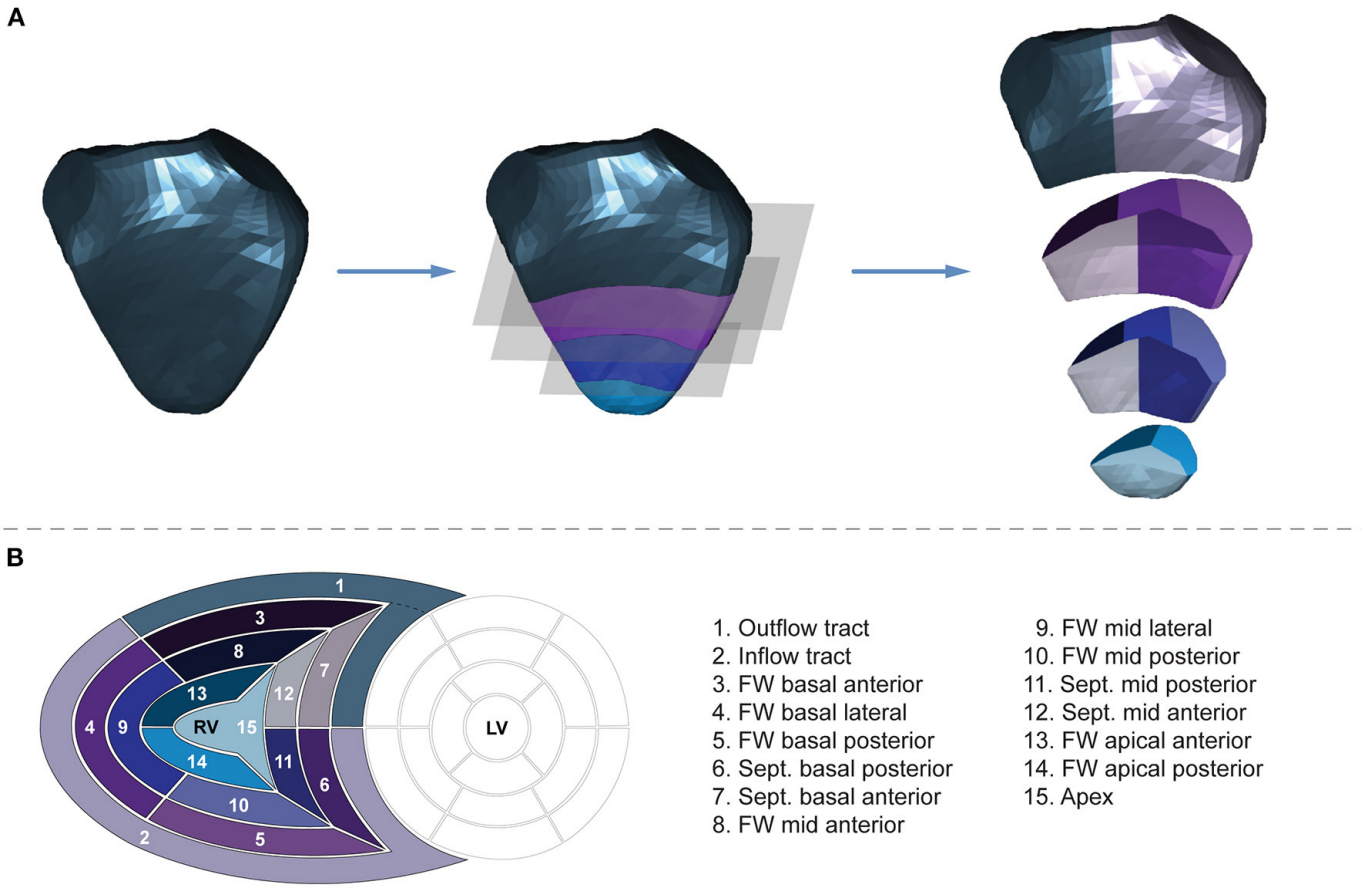
Volumetric partitioning of the right ventricle. **(A)** Segmentation is performed on the end-diastolic mesh of each series to obtain 15 segments. First, the fraction of the mesh containing the inflow and outflow segments is separated by a horizontal slicing plane positioned at a predefined height along the longitudinal (i.e., vertical) axis. Then, the remainder of the mesh is trisected by two other horizontal planes at equidistant heights. Next, the horizontal slices are divided further. The inflow segment is separated from the outflow segment by a vertical slicing plane along the midpoint of the central vertices of the tricuspid and pulmonary annuli. Using vertically aligned standard planes, the septal and free wall portions of the horizontal slices are divided into further segments. The vertical slicing planes split the slices into three free wall and two septal segments on the basal and mid-levels and into two free wall and one apical segments on the apical level aiming for equal volume distribution among the segments within the given slice. See text for further details. The 3D right ventricular model is visualized from septal point of view. **(B)** The “bull's eye” plot and the nomenclature of the 15 newly generated right ventricular segments. FW, free wall; LV, left ventricle; RV, right ventricle; Sept., septum.

Volumetric segmentation is performed on the end-diastolic mesh of each series to obtain 15 RV segments (Figure 3). First, the fraction of the mesh containing the inflow and outflow segments is separated by a horizontal slicing plane positioned at a predefined height along the longitudinal (i.e., vertical) axis (*d*_1_–a vector defining the height of the slicing plane along the vertical axis). Then, the remainder of the mesh is trisected by two other horizontal planes at equidistant heights. The following parametric equation represents each horizontal slicing plane:

$$\begin{array}{l} a^{T}n_{i}+\;d_{i}=0 \end{array}$$

where the *n*_1_ = *n*_2_ = *n*_3_ normal vectors of the slicing planes are vertical, and *d*_3_ and *d*_2_ are placed at equidistant heights between *d*_1_ and the most apical point of the RV (i.e., the vertex with the minimal y-coordinate).

Next, the horizontal slices are divided further. The inflow segment is separated from the outflow segment by a vertical slicing plane along the midpoint of the central vertices of the tricuspid and pulmonary annuli. Using vertically aligned standard planes, the septal and free wall portions of the horizontal slices are divided into further segments. The vertical slicing planes split the slices into three free wall and two septal segments on the basal and mid-levels and into two free wall and one apical segments on the apical level aiming for equal volume distribution among the segments within the given slice. After the parcellation is completed, the newly generated (non-closed) sides of the segments are covered by smooth biharmonic surfaces. As the segmentation is performed on the end-diastolic mesh, the positions of the newly generated vertices are interpolated in all other frames using their barycentric coordinates.

#### Calculation of Global and Segmental Metrics and Quantifying the Relative Contribution of Longitudinal, Radial, and Anteroposterior Motion Components

To calculate global longitudinal strain (GLS), 45 longitudinally oriented contours (i.e., longitudes) are generated by connecting the apex (*v*_*a*_) and the predefined vertices of the RV base ($E=\;{\left\{v_{e}^{\left(k\right)}\right\}}_{k=1}^{n}$) through specific vertices of the middle section of the RV ($C=\;{\left\{v_{c}^{\left(k\right)}\right\}}_{k=1}^{n}$) with geodesic lines. This method ensures that the longitudes are distributed evenly on the surface of the mesh. The length of the *j*^*th*^ longitude ($L_{j}^{i}$) can be calculated as the sum of the *v*_*a*_-$v_{c}^{\left(k\right)}$ and $v_{c}^{\left(k\right)}$-$v_{e}^{\left(k\right)}$ geodesic distances. The change in the length of each longitude can be monitored throughout the entire cardiac cycle, and GLS can be computed using the following formula:

$$\begin{array}{l} GLS\;\left(\%\right)=100\;*\;\overset{n}{\underset{j=1}{\sum }}\left(\frac{L_{j}^{end-systole}}{L_{j}^{end-diastole}}-1\right) \end{array}$$

For global circumferential strain (GCS) calculations, the inflow and outflow segments are omitted. Fifteen circumferential contours (i.e., latitudes) are created by slicing the mesh with horizontal planes at equal distances along the longitudinal axis. After generating the set of circumferential contours $[{\left\{C_{j}^{\left(i\right)}\right\}}_{j=1\ldots n}$, where $C_{j}^{\left(i\right)}=\left(v_{1},v_{2},\ldots ,\;v_{l}\right)$ is the list of vertices on a single contour], the length of the *j*^*th*^ circumferential contour is computed as:

$$\begin{array}{l} C_{j}^{i}=\overset{l-1}{\underset{i=1}{\sum }}||v_{i+1}-v_{i}|| \end{array}$$

GCS is calculated using the contour lengths at end-diastole and end-systole:

$$\begin{array}{l} GCS\;\left(\%\right)=100\;*\;\overset{n}{\underset{j=1}{\sum }}\left(\frac{C_{j}^{end-systole}}{C_{j}^{end-diastole}}-1\right) \end{array}$$

Global area strain (GAS) quantifies the change in the endocardial surface area between end-diastolic and end-systolic frames. The surface area of the ${\hat{m}}_{i}$ triangle mesh can be assessed as:

$$\begin{array}{l} A^{i}=\;\underset{t\in T^{i}}{\sum }\frac{\left||\left(t_{1}-t_{3}\right)\times \left(t_{2}-t_{3}\right)|\right|}{2} \end{array}$$

where $T^{i}=\;{\left\{t_{k}\in R^{3}\right\}}_{k=1\ldots }$ is the set of triangles of the ${\hat{m}}_{i}\;$ mesh.

Similar to previous calculations, GAS is defined as:

$$\begin{array}{l} GAS\;\left(\%\right)=100\;*\;\left(\frac{A^{end-systole}}{A^{end-diastole}}-1\right) \end{array}$$

The volume of each mesh (or segment) is calculated using the shoelace formula (19). Let *T* be the set of triangles ${\hat{m}}_{i}$ mesh. For each *t* = {(*x*_1_, *y*_1_, *z*_1_), (*x*_2_, *y*_2_, *z*_2_), (*x*_3_, *y*_3_, *z*_3_)}∈*T*, the volume of the tetrahedron bounded by the vertices of *t* and the origin:

$$\begin{array}{l} V_{t}:=\frac{1}{6}\left(-x_{3}y_{2}z_{1}+x_{2}y_{3}z_{1}+x_{3}y_{1}z_{2}-x_{1}y_{3}z_{2}-x_{2}y_{1}z_{3}+x_{1}y_{2}z_{3}\right) \end{array}$$

Note that *V*_*t*_ is signed, meaning that its value may be negative if the normal vector of the triangle points toward the origin. According to the signed tetrahedron method, the volume of the ${\hat{m}}_{i}$ mesh is the sum of the signed *V*_*t*_ volumes:

$$\begin{array}{l} V=\underset{t\in T}{\sum }V_{t} \end{array}$$

Motion decomposition is performed along the aforementioned directions in a vertex-based manner, as previously described (9). End-systolic volumes (ESV) and corresponding EFs generated by each motion component (longitudinal ESV and EF, radial ESV and EF, anteroposterior ESV and EF) can be quantified.

Beyond the global parameters, we can calculate regional metrics (i.e., septal and free wall longitudinal or area strains; basal-, mid-, and apical-level circumferential or area strains) and also segmental metrics for each of the 15 segments (segmental strains and volumes).

### Reproducibility of Global and Segmental RV Metrics

Although the second, the third, and the fourth steps of the ReVISION pipeline (i.e., the orientation adjustment, the motion decomposition, the volumetric segmentation, and the calculation of metrics) are fully automated, and they do not introduce any additional observer-related variability, we sought to analyze how the differences in 3D contouring and 3D model reconstruction (using the dedicated TomTec software solution) affect the results of our analysis.

#### Study Population

To assess the reproducibility of global and segmental RV metrics, 10 healthy, sedentary control subjects (five males, 21 ± 2 years), 10 elite water polo athletes (five males, 21 ± 5 years, 22 ± 4 h of training per week), and 10 end-stage heart failure patients with reduced LV EF (seven males, 57 ± 14 years) were retrospectively identified in our database. Thus, subjects represented a wide range of cardiac volumes and function. The study protocol conforms with the principles outlined in the Declaration of Helsinki and the local regulatory and data protection standards (20). All subjects in our database were enrolled as part of prospective studies (each approved by the Regional and Institutional Committee of Science and Research Ethics, approval no. 13687-0/2011-EKU and 034309-006/2014/OTIG) and provided written informed consent prior to enrollment to the archiving and analysis of their datasets and the publication of subsequent results.

#### 3D Echocardiography

Echocardiographic examinations were performed with a commercially available ultrasound system (GE Vivid E95, 4Vc-D probe, Horten, Norway) in all cases. Beyond the conventional echocardiographic protocol, ECG-gated full-volume 3D datasets reconstructed from four cardiac cycles and optimized for the RV were obtained from apical view targeting a minimum volume rate of 25 volumes/second. Datasets were processed using a commercially available dedicated software solution (4D RV-Function 2, TomTec Imaging, Unterschleissheim, Germany), and RV end-diastolic volume (EDV), ESV, EF, 2D free wall, and septal longitudinal strain were measured. The 3D models of the RV were exported frame by frame throughout the cardiac cycle for further analysis with the ReVISION method.

#### Analyzing Intra- and Interobserver Reproducibility

To assess the intraobserver reproducibility of the parameters, the operator (BKL), who performed the first measurements, repeated the 3D reconstruction (using TomTec 4D RV-Function 2) and analysis of the RV models blinded to previous results. Then, a second experienced operator (AK) also performed the 3D reconstruction (with TomTec 4D RV-Function 2) and the ReVISION analysis of the same subjects in a blinded fashion in order to determine interobserver reproducibility.

#### Comparison of ReVISION- and TomTec-Derived RV Longitudinal Strains

Using the first series of measurements performed by the original operator (BKL), the correlations were assessed between RV longitudinal strain values computed with the ReVISION method (3D global, free wall, and septal longitudinal strain) and the TomTec 4D RV-Function 2 (2D free wall and septal longitudinal strain). In the calculation of RV longitudinal strains, an important technical difference should be noted between the two software solutions: the TomTec 4D RV-Function 2 assesses 2D free wall and septal longitudinal strains using 2D standard apical four-chamber views derived from the 3D datasets, whereas the ReVISION method calculates 3D global, free wall, and septal longitudinal strains using the reconstructed 3D meshes of the RV, as described above.

#### Statistical Analysis

The intra- and interobserver variability and reliability were evaluated using the intraclass correlation coefficient and the coefficient of variation, respectively. The correlations between the ReVISION- and TomTec-derived longitudinal strain measurements were quantified using Pearson correlation coefficients. A *p* < 0.05 was considered significant. All statistical analyses were performed in R (version 3.6.2, R Foundation for Statistical Computing, Vienna, Austria).

### Comparison of 3DE- and CMRI-Derived Metrics

#### Study Population

Six healthy, sedentary control subjects (three males, 21 ± 2 years) without any known cardiovascular disease or risk factors, along with six healthy elite athletes of various sports disciplines (four males, 23 ± 8 years, 15 ± 6 h of training per week) and six heart failure patients with reduced LV EF in a stable clinical and hemodynamic condition (five males, 73 ± 7 years) were retrospectively identified in our database to match our predefined criterion of having a 3DE and a CMRI examination within 30 days. This population was used to investigate the agreement between the 3DE- and CMRI-based 3D RV models concerning the relative contribution of longitudinal, radial, and anteroposterior motion components. All analyses and measurements were performed blinded to the results assessed with the other imaging modality. The study protocol conforms with the principles outlined in the Declaration of Helsinki and the local regulatory and data protection standards (20). All subjects in our database were enrolled as part of prospective studies (each approved by the Regional and Institutional Committee of Science and Research Ethics, approval no. 13687-0/2011-EKU and 034309-006/2014/OTIG) and provided written informed consent prior to enrollment to the archiving and analysis of their datasets and the publication of subsequent results.

#### 3D Echocardiography

Echocardiographic examinations, 3D model reconstruction, and analyses were performed in the same way as explained in the previous section (*Reproducibility of Global and Segmental RV Metrics*) of this paper.

#### CMRI Protocol

CMRI examinations were conducted using a 1.5-Tesla MRI scanner (Achieva, Philips Medical Systems, Eindhoven, The Netherlands) with a five-channel cardiac coil. Retrospectively gated, balanced steady-state free precession cine images were acquired in conventional long- and short-axis views covering the LV and RV. Short-axis cine images were obtained with 8-mm slice thickness (no interslice gaps), in-plane resolution of 1.5 × 1.5 mm and temporal resolution of 25 phases per cardiac cycle.

#### Reconstruction of 3D RV Meshes From CMR Images

**Figure 4:**
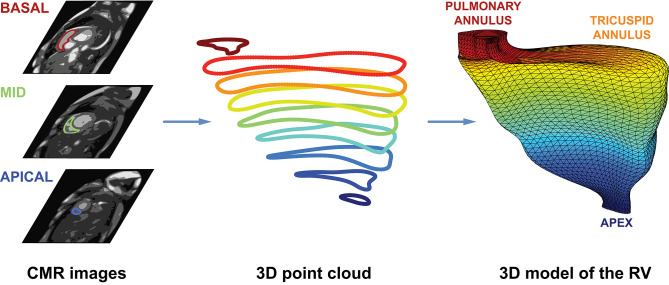
Schematic illustration of the CMRI-based reconstruction algorithm. After the end-diastolic and end-systolic cardiac phases were identified, the epi- and endocardial layers were manually traced in cine short-axis images. Then, the endocardial contours of the right ventricle were exported to separate files as a series of 2D point coordinates. These files were supplied into a 3D mesh reconstruction pipeline that comprises four consecutive steps: (i) preprocessing contour data, (ii) creating 3D point clouds from contour data, (iii) reindexing the vertices in the 3D point clouds, and (iv) fitting closed surfaces to the 3D point clouds. See text for further details. CMR, cardiac magnetic resonance; RV, right ventricle.

After the end-diastolic and end-systolic cardiac phases were identified, the epi- and endocardial layers were manually traced in cine short-axis images, and RV EDV, ESV, and EF were assessed with a dedicated post-processing software solution (Medis Qmass 7.6, Medis, Leiden, The Netherlands). The endocardial contours of the RV were exported to separate files as a series of 2D point coordinates. These files were supplied into a 3D mesh reconstruction pipeline that comprises four consecutive steps: (i) preprocessing contour data, (ii) creating 3D point clouds from contour data, (iii) reindexing the vertices in the 3D point clouds, and (iv) fitting closed surfaces to the 3D point clouds (Figure 4).

In the preprocessing step, a cubic B-spline (containing exactly 200 points) was fitted to the 2D contour points of each slice.

To create a 3D object from the 2D contours, point coordinates were converted to millimeters, and the z coordinates were also generated based on the position of the slice along the vertical axis of the RV. The slices were ordered; therefore, the first slice was always the closest to the apex (*z* = 0), and each slice is located 8 mm above the previous one. By convention, the center of each point cloud (i.e., set of contour points) was shifted to the origin.

**Figure 5:**
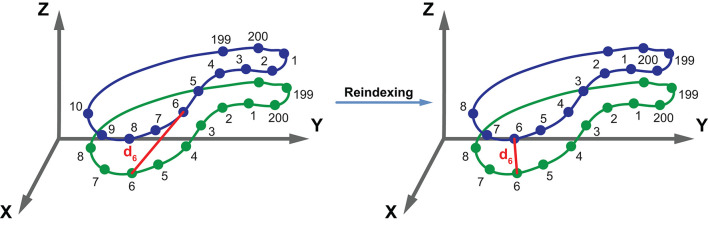
Schematic illustration of the reindexing step. The green and blue contours represent two slices containing equal number (*n* = 200) of points. The vertices of the blue contour are reindexed in a way that the mean squared error between the corresponding vertices of the green and blue contours becomes minimal. With appropriate reindexing, a vertex with a given index is located approximately at the same anatomical position in both contours. See text for further details. d_6_, the distance between the points with the index 6 of the two contours.

As the next step, the 3D points (i.e., vertices) in the point clouds were reindexed. With appropriate reindexing, a vertex with a given index was located approximately at the same anatomical position in the end-diastolic and end-systolic phases, which was essential to perform the motion decomposition. First, the middle slice of the end-systolic point cloud was reindexed in a way that the mean squared error between the corresponding vertices of the end-diastolic and end-systolic slices became minimal:

$$\begin{array}{l} I^{*}=\arg\;\underset{I}{\min}\underset{k}{\sum }||{\vec{R}}_{k}-{\vec{S}}_{I\;\left(k\right)}|{|}_{2}^{2} \end{array}$$

where *R* is the reference slice (i.e., the middle slice of the end-diastolic point cloud), *S* is the slice that is being reindexed (i.e., the middle slice of the end-systolic point cloud), and *I* is the function for reindexing that returns the corresponding index of the contour point in *S* for the *k*^*th*^ point of *R* (Figure 5). Then, the same method was applied to reassign the indices of the remaining slices within each point cloud using the previously reindexed middle slice as a reference.

Preceding the surface fitting, duplicates of the most basal and apical slices were placed 4 mm above and below the original ones. Then, a closed triangle mesh was fitted to the vertices using the surface interpolation algorithm as implemented in the *geomdl* Python library (version 5.2.10) (21). As a result, the slices were covered by a triangle mesh; however, the top and bottom remained opened. To close the top and bottom of the mesh, constrained Delaunay triangulation was performed, ensuring that the triangulation is successful even if the shape of the given object is not convex (22). Finally, the volume of each mesh was calculated using the shoelace formula.

The entire 3D mesh reconstruction pipeline was implemented in Python (version 3.8.2, Python Software Foundation, Wilmington, Delaware, USA).

To validate the reconstruction process, the RV EDV, ESV, and EF derived from the reconstructed meshes were compared to those computed using the dedicated post-processing software. An excellent agreement was observed in all three metrics (Supplementary Figure 1).

#### Comparison of 3DE- and CMRI-Derived Metrics

To enable the comparison of 3DE- and CMRI-derived metrics within the same patient, we had to ensure that the meshes are aligned in the same orientation. To that end, 3DE- and CMRI-derived end-diastolic meshes were visualized, and their orientation was adjusted manually by an experienced operator (MT) to match the orientation of the 3DE-derived mesh. Then, the rotation matrix was extracted and was applied to the end-systolic CMRI mesh of the same patient as well. After adjusting the orientation of the CMRI-derived meshes, their motion was decomposed as described previously (9).

The correlations between 3DE-derived measurements and the corresponding CMRI-derived values were quantified using Pearson correlation coefficients, and Bland–Altman analyses were performed to assess the bias and limits of agreement. Paired Wilcoxon signed-rank test vs. null values was applied to test the significance of the bias. A p < 0.05 was considered significant. All statistical analyses were performed in R (version 3.6.2, R Foundation for Statistical Computing, Vienna, Austria).

## Results

### Reproducibility of Global and Segmental RV Metrics

**Figure 6:**
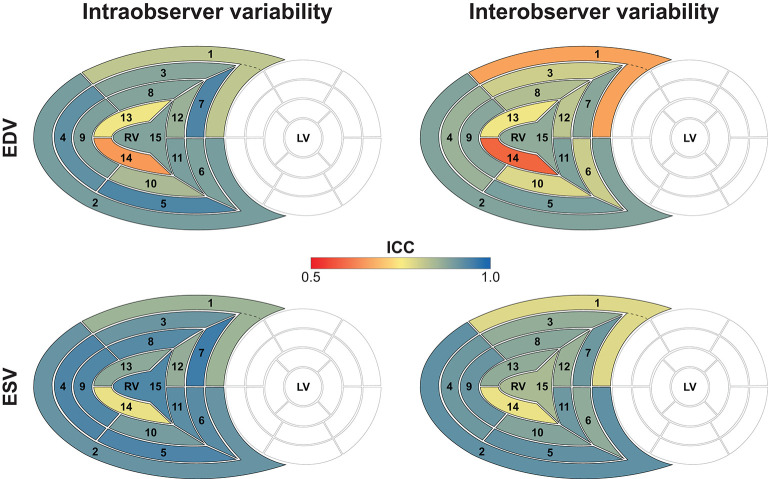
Intra- and interobserver variability of right ventricular end-diastolic and end-systolic segmental volumes. The 15 segments are color-coded based on the value of the intraclass correlation coefficient: reddish colors indicate a lower, whereas blueish colors correspond to a higher value of intraclass correlation coefficient in the given segment. Segment numbers as defined in Figure 3. EDV, end-diastolic volume; ESV, end-systolic volume; ICC, intraclass correlation coefficient; LV, left ventricle; RV, right ventricle.

**Figure 7:**
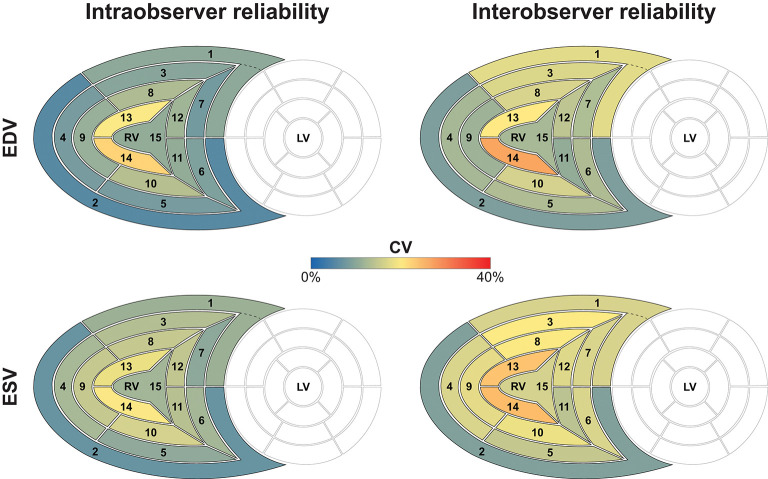
Intra- and interobserver reliability of right ventricular end-diastolic and end-systolic segmental volumes. The 15 segments are color-coded based on the value of the coefficient of variation: reddish colors indicate a higher, whereas blueish colors correspond to a lower value of coefficient of variation. Segment numbers as defined in Figure 3. EDV, end-diastolic volume; ESV, end-systolic volume; CV, coefficient of variation; LV, left ventricle; RV, right ventricle.

The results of the intra- and interobserver variability and reliability analyses are summarized in Tables 1, 2. Reproducibility of global EDV, ESV, and decomposed ESVs were high, which is consistent with our previous reports using the earlier versions of the ReVISION software (10, 11, 13). Regarding the 15 segments, a smooth base-to-apex gradient could be observed (Table 2, Figures 6, 7), with the inflow tract and basal segments having the lowest variability and the highest reliability and free wall apical segments exhibiting the highest variability and the lowest reliability. As the orientation adjustment, the motion decomposition, the volumetric segmentation, and the calculation of metrics are fully automated, it should be emphasized that the observed intra- and interobserver variability is related exclusively to the 3D RV model reconstruction that is performed using TomTec 4D RV-Function 2.

**Table 1:** 3D echocardiographic parameters measured by the two operators in the reproducibility analysis.

	**Operator 1**		**Operator 2**
	**1st measurements**	**2nd measurements**	
3D RV EDV, mL	167.5 ± 63.1	174.2 ± 66.3	183.2 ± 64.3
3D RV ESV, mL	95.4 ± 55.7	98.4 ± 60.6	99.8 ± 56.2
LESV, mL	134.2 ± 58.6	140.8 ± 63.5	144.9 ± 60.5
RESV, mL	131.2 ± 55.2	135.3 ± 59.5	143.4 ± 58.4
AESV, mL	130.9 ± 62.8	135.9 ± 66.7	141.0 ± 62.9
3D RV GLS, %	−17.6 ± 7.1	−18.5 ± 7.9	−18.4 ± 7.5
3D RV GCS, %	−19.5 ± 9.0	−20.7 ± 9.4	−19.4 ± 7.5
3D RV GAS, %	−31.6 ± 12.2	−32.8 ± 13.2	−33.2 ± 12.1
Outflow tract EDV, mL	47.9 ± 19.0	51.5 ± 21.0	55.9 ± 20.4
Outflow tract ESV, mL	29.5 ± 15.9	31.6 ± 18.3	31.8 ± 17.1
Inflow tract EDV, mL	33.9 ± 12.5	35.2 ± 13.7	34.4 ± 12.8
Inflow tract ESV, mL	22.8 ± 11.2	24.5 ± 13.3	22.9 ± 12.8
FW basal anterior EDV, mL	14.3 ± 7.4	14.5 ± 6.5	16.0 ± 6.3
FW basal anterior ESV, mL	7.1 ± 5.6	6.8 ± 5.3	7.3 ± 4.7
FW basal lateral EDV, mL	11.7 ± 5.0	11.7 ± 4.5	12.3 ± 4.4
FW basal lateral ESV, mL	5.3 ± 4.0	5.2 ± 3.9	5.5 ± 3.6
FW basal posterior EDV, mL	8.5 ± 3.9	8.5 ± 4.0	8.4 ± 3.5
FW basal posterior ESV, mL	4.5 ± 3.2	4.5 ± 3.4	4.6 ± 3.2
Sept. basal posterior EDV, mL	8.3 ± 3.4	8.5 ± 3.2	9.2 ± 3.3
Sept. basal posterior ESV, mL	4.4 ± 3.0	4.2 ± 2.9	4.5 ± 2.7
Sept. basal anterior EDV, mL	6.5 ± 2.8	6.6 ± 2.7	6.7 ± 2.6
Sept. basal anterior ESV, mL	3.6 ± 2.4	3.6 ± 2.4	3.7 ± 2.3
FW mid anterior EDV, mL	8.5 ± 4.5	8.8 ± 4.2	9.8 ± 4.3
FW mid anterior ESV, mL	4.1 ± 3.4	3.8 ± 3.2	4.3 ± 2.9
FW mid lateral EDV, mL	7.3 ± 3.1	7.4 ± 3.0	7.9 ± 3.2
FW mid lateral ESV, mL	3.2 ± 2.3	3.1 ± 2.3	3.4 ± 2.2
FW mid posterior EDV, mL	3.8 ± 1.7	3.8 ± 1.7	3.8 ± 1.8
FW mid posterior ESV, mL	1.9 ± 1.3	1.9 ± 1.4	1.9 ± 1.4
Sept. mid posterior EDV, mL	5.2 ± 2.2	5.4 ± 2.3	5.8 ± 2.5
Sept. mid posterior ESV, mL	2.7 ± 1.9	2.7 ± 1.9	2.9 ± 1.8
Sept. mid anterior EDV, mL	3.5 ± 1.4	3.6 ± 1.4	3.7 ± 1.5
Sept. mid anterior ESV, mL	1.9 ± 1.3	1.9 ± 1.2	2.0 ± 1.2
FW apical anterior EDV, mL	2.1 ± 1.3	2.2 ± 1.3	2.5 ± 1.4
FW apical anterior ESV, mL	1.1 ± 1.0	1.1 ± 1.1	1.2 ± 1.0
FW apical posterior EDV, mL	1.1 ± 0.6	1.1 ± 0.5	1.2 ± 0.7
FW apical posterior ESV, mL	0.5 ± 0.4	0.5 ± 0.4	0.6 ± 0.4
Apex EDV, mL	5.1 ± 2.5	5.2 ± 2.3	5.5 ± 2.8
Apex ESV, mL	3.0 ± 2.1	2.9 ± 2.1	3.2 ± 2.2

*Cell values are mean ± standard deviation*.

*AESV, anteroposterior end-systolic volume; EDV, end-diastolic volume; ESV, end-systolic volume; FW, free wall; GAS, global area strain; GCS, global circumferential strain; GLS, global longitudinal strain; LESV, longitudinal end-systolic volume; RESV, radial end-systolic volume; RV, right ventricular; Sept., septum*.

**Table 2:** Intra- and interobserver variability and reliability of global and segmental right ventricular metrics.

	**Intraobserver reproducibility**		**Interobserver reproducibility**	
	**ICC (95% CI)**	**CV (%)**	**ICC (95% CI)**	**CV (%)**
3D RV EDV	0.945 (0.898–0.970)	5.840	0.902 (0.758–0.953)	9.298
3D RV ESV	0.955 (0.918–0.975)	6.529	0.947 (0.904–0.971)	9.131
LESV	0.943 (0.895–0.969)	6.525	0.920 (0.836–0.959)	9.471
RESV	0.938 (0.889–0.966)	6.514	0.895 (0.785–0.946)	10.333
AESV	0.952 (0.912–0.974)	6.761	0.932 (0.862–0.965)	10.322
3D RV GLS	0.937 (0.881–0.966)	9.296	0.911 (0.841–0.951)	12.240
3D RV GCS	0.944 (0.892–0.971)	11.588	0.845 (0.731–0.913)	18.011
3D RV GAS	0.972 (0.946–0.985)	5.995	0.951 (0.902–0.975)	8.271
Outflow tract EDV	0.818 (0.686–0.898)	10.448	0.674 (0.422–0.819)	17.711
Outflow tract ESV	0.854 (0.745–0.918)	11.670	0.787 (0.639–0.879)	16.414
Inflow tract EDV	0.895 (0.815–0.942)	5.889	0.883 (0.793–0.935)	9.119
Inflow tract ESV	0.919 (0.855–0.956)	7.357	0.914 (0.843–0.953)	9.414
FW basal anterior EDV	0.878 (0.786–0.932)	9.999	0.803 (0.645–0.892)	16.974
FW basal anterior ESV	0.907 (0.834–0.949)	13.648	0.864 (0.763–0.924)	19.973
FW basal lateral EDV	0.919 (0.856–0.956)	8.499	0.856 (0.749–0.920)	12.475
FW basal lateral ESV	0.938 (0.888–0.966)	12.626	0.904 (0.829–0.947)	16.693
FW basal posterior EDV	0.937 (0.887–0.966)	8.528	0.892 (0.810–0.940)	13.269
FW basal posterior ESV	0.940 (0.892–0.967)	10.607	0.918 (0.853–0.955)	14.988
Sept. basal posterior EDV	0.888 (0.802–0.938)	9.302	0.806 (0.650–0.893)	13.187
Sept. basal posterior ESV	0.926 (0.868–0.960)	12.383	0.868 (0.769–0.927)	16.650
Sept. basal anterior EDV	0.938 (0.890–0.966)	8.321	0.876 (0.782–0.931)	13.294
Sept. basal anterior ESV	0.953 (0.914–0.974)	10.262	0.904 (0.829–0.947)	16.537
FW mid anterior EDV	0.881 (0.790–0.934)	13.051	0.833 (0.659–0.914)	16.636
FW mid anterior ESV	0.929 (0.872–0.961)	15.034	0.876 (0.782–0.931)	19.886
FW mid lateral EDV	0.887 (0.800–0.937)	11.484	0.877 (0.773–0.933)	11.708
FW mid lateral ESV	0.935 (0.884–0.965)	15.028	0.911 (0.842–0.951)	17.802
FW mid posterior EDV	0.840 (0.723–0.910)	13.812	0.790 (0.644–0.881)	16.231
FW mid posterior ESV	0.897 (0.817–0.943)	16.277	0.881 (0.790–0.933)	18.573
Sept. mid posterior EDV	0.893 (0.810–0.930)	11.051	0.886 (0.724–0.945)	11.087
Sept. mid posterior ESV	0.924 (0.864–0.958)	13.877	0.906 (0.832–0.948)	14.244
Sept. mid anterior EDV	0.859 (0.754–0.921)	12.028	0.820 (0.691–0.890)	14.455
Sept. mid anterior ESV	0.865 (0.764–0.925)	14.670	0.859 (0.754–0.921)	17.253
FW apical anterior EDV	0.764 (0.604–0.866)	19.717	0.764 (0.573–0.871)	20.048
FW apical anterior ESV	0.872 (0.769–0.930)	18.407	0.867 (0.768–0.927)	23.412
FW apical posterior EDV	0.664 (0.456–0.803)	22.065	0.608 (0.381–0.767)	25.802
FW apical posterior ESV	0.772 (0.615–0.870)	19.669	0.771 (0.601–0.872)	24.043
Apex EDV	0.883 (0.789–0.936)	11.067	0.868 (0.769–0.927)	12.545
Apex ESV	0.936 (0.885–0.965)	12.204	0.841 (0.725–0.911)	16.263

*CI, confidence interval; CV, coefficient of variation; ICC, intraclass correlation coefficient*.

*Other abbreviations as in Table 1*.

Three-dimensional GLS correlated robustly with both 2D free wall and septal longitudinal strains (*r* = 0.907 and *r* = 0.891, both *p* < 0.001, Table 3). When we analyzed free wall and septal longitudinal strains separately with our software solution, both 3D free wall and septal longitudinal strains showed strong correlations with the corresponding TomTec-derived values (*r* = 0.920 and *r* = 0.919, both *p* < 0.001, Table 3).

**Table 3:** Correlations between the ReVISION- and TomTec-derived right ventricular strains (*n* = 30).

		**TomTec**	
		**2D free wall longitudinal strain**	**2D septal longitudinal strain**
**ReVISION**	**3D GLS**	*r* = 0.907 *p* < 0.001	*r* = 0.891 *p* < 0.001
	**3D free wall longitudinal strain**	*r* = 0.920 *p* < 0.001	–
	**3D septal longitudinal strain**	–	*r* = 0.919 *p* < 0.001

*Statistical test: Pearson correlation*.

*GLS, global longitudinal strain*.

### Comparison of 3DE- and CMRI-Derived Metrics

**Figure 8:**
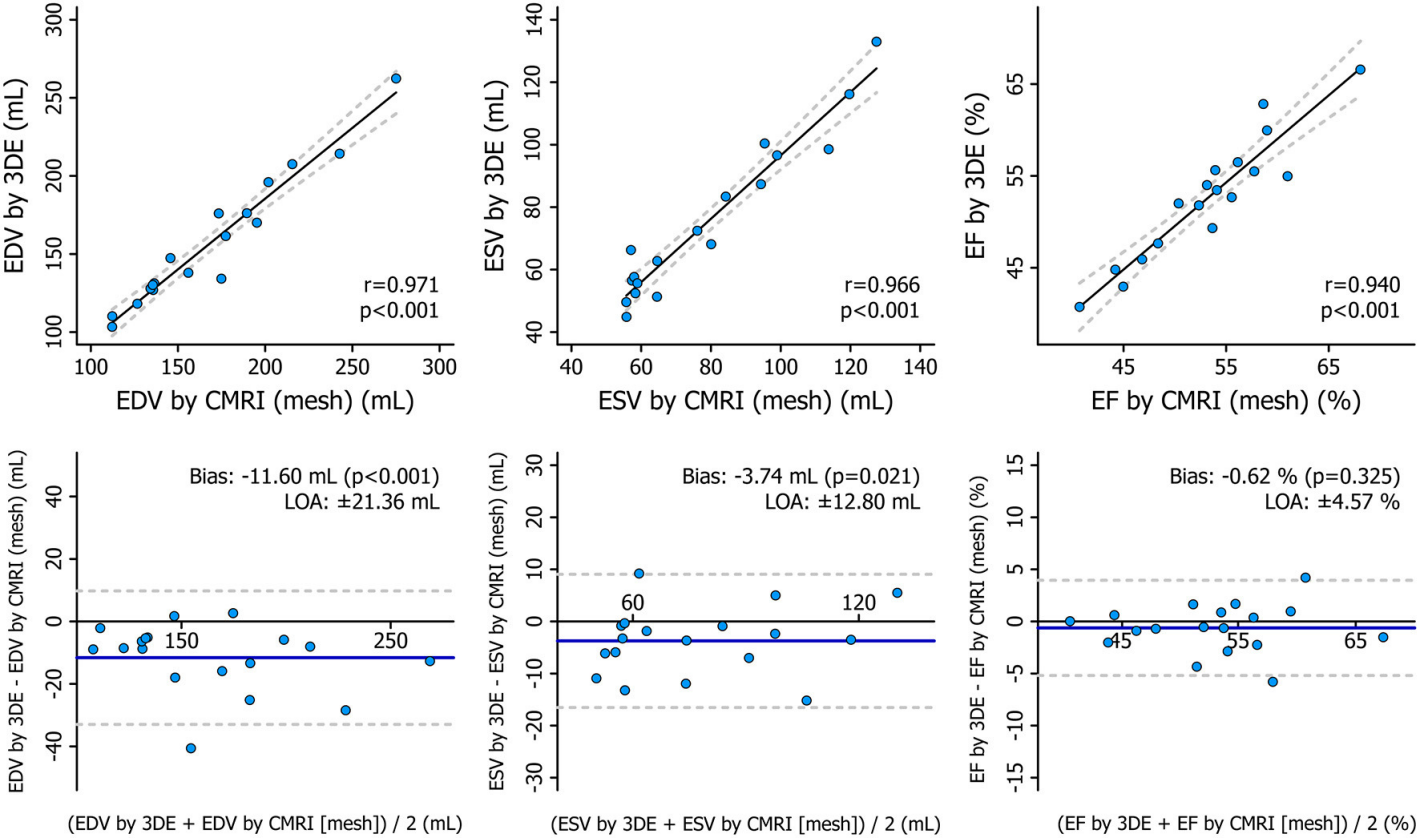
Correlation and agreement between 3D echocardiography- and cardiac magnetic resonance imaging-derived measurements of right ventricular end-diastolic volume, end-systolic volume, and ejection fraction depicted by correlation and Bland–Altman plots. The correlations between 3D echocardiography-derived measurements and the corresponding cardiac magnetic resonance imaging-derived values were quantified using Pearson correlation coefficients, and Bland–Altman analyses were performed to assess the bias and limits of agreement. Paired Wilcoxon signed-rank test vs. null values was applied to test the significance of the bias. 3DE, 3D echocardiography; CMRI, cardiac magnetic resonance imaging; EDV, end-diastolic volume; EF, ejection fraction; ESV, end-systolic volume; LOA, limits of agreement.

**Figure 9:**
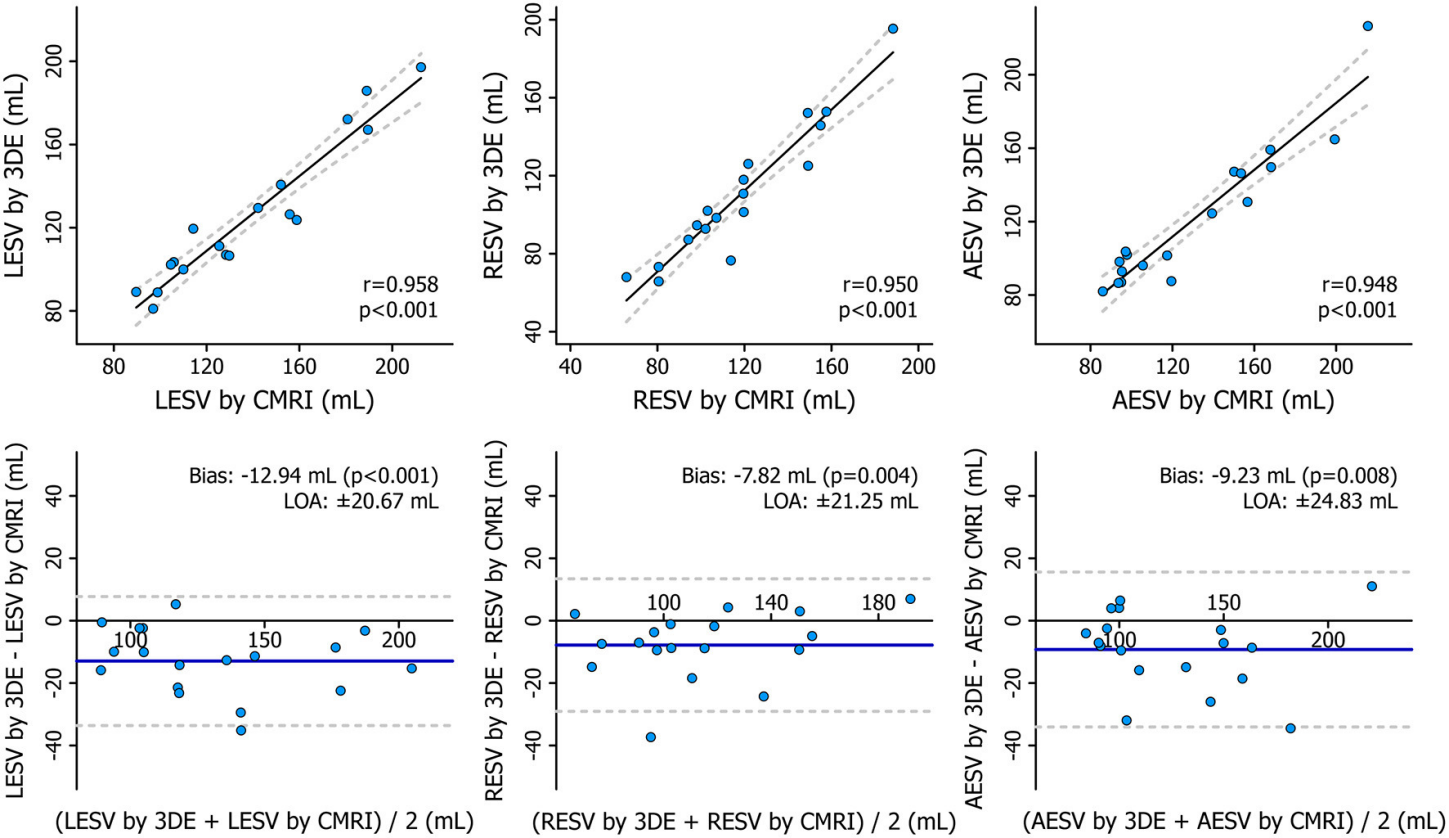
Correlation and agreement between 3D echocardiography- and cardiac magnetic resonance imaging-derived measurements of right ventricular decomposed end-systolic volumes depicted by correlation and Bland–Altman plots. Statistical tests: same as in Figure 8. 3DE, 3D echocardiography; AESV, anteroposterior end-systolic volume; CMRI, cardiac magnetic resonance imaging; LESV, longitudinal end-systolic volume; LOA, limits of agreement; RESV, radial end-systolic volume.

**Figure 10:**
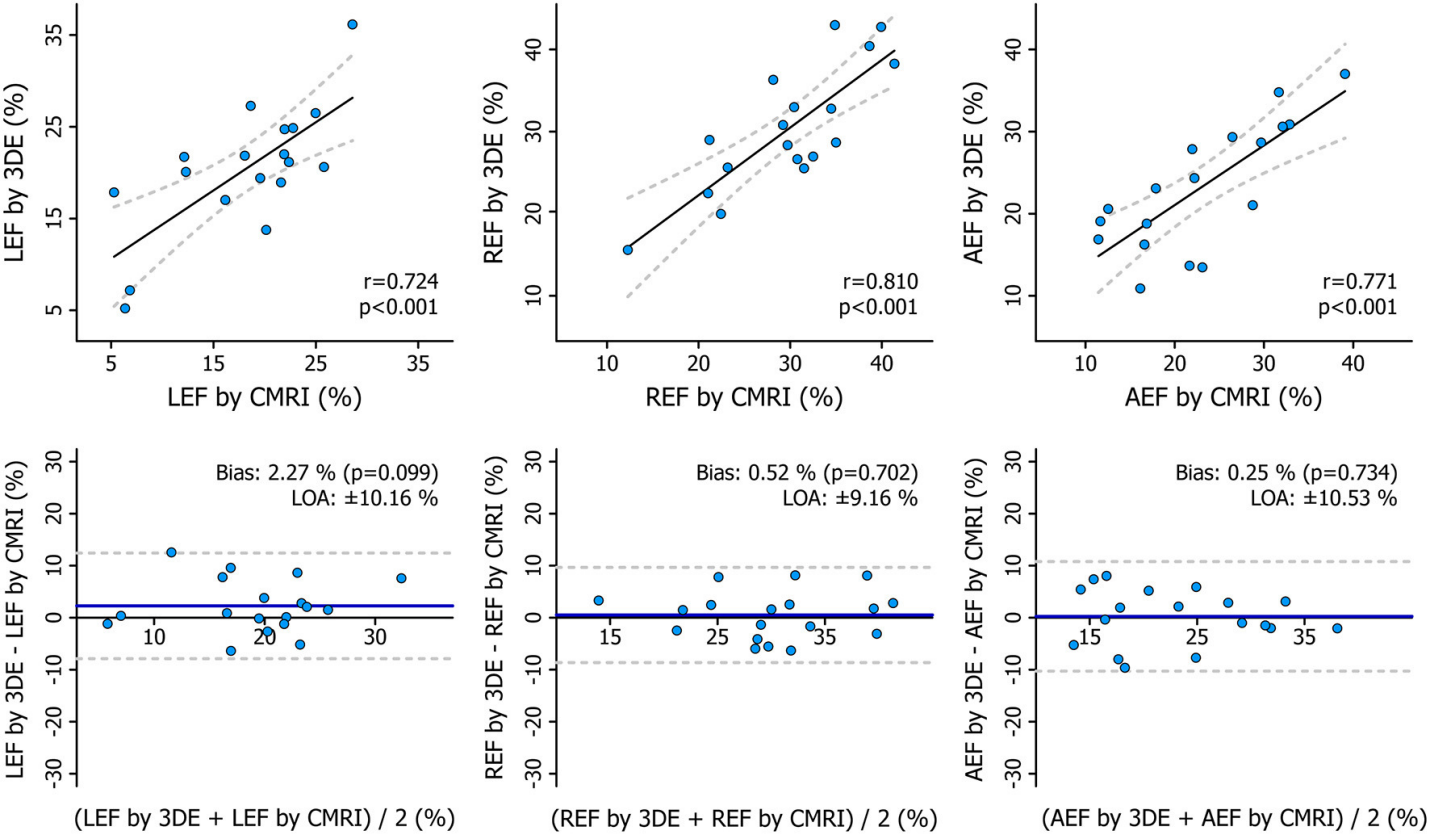
Correlation and agreement between 3D echocardiography- and cardiac magnetic resonance imaging-derived measurements of right ventricular decomposed ejection fractions depicted by correlation and Bland–Altman plots. Statistical tests: same as in Figure 8. 3DE, 3D echocardiography; AEF, anteroposterior ejection fraction; CMRI, cardiac magnetic resonance imaging; LEF, longitudinal ejection fraction; LOA, limits of agreement; REF, radial ejection fraction.

The mean values of the measurements performed with the two imaging modalities are presented in Table 4. 3DE- and CMRI-derived volumes correlated robustly, and a systematic underestimation by 3DE could be seen (Figure 8, Table 5), which is in line with previous studies (23, 24). RV EF demonstrated a good correlation, and only a negligible bias could be observed between its values derived from the two imaging modalities (Table 5). The comparison of 3DE- and CMRI-derived ESVs after motion decomposition along the three aforementioned directions is illustrated in Figure 9. Strong correlations could be reported (Pearson correlation coefficients > 0.9 of the three motion components), and similar to global RV volumes, a consistent underestimation of volumes by 3DE could also be detected in the values of the decomposed volumes (Table 5). Not surprisingly, the values of the decomposed EFs showed slightly weaker but highly significant correlations between the two modalities, and in addition, bias was found to be non-significant in all of them (Figure 10, Table 5).

**Table 4:** Summary of the 3D echocardiography- and cardiac magnetic resonance imaging-derived measurements.

	**Healthy controls *n* = 6**	**Athletes *n* = 6**	**HFrEF patients *n* = 6**
**3D ECHOCARDIOGRAPHY**			
RV EDV, mL	140.7 ± 19.9	185.4 ± 52.6	145.8 ± 37.6
RV ESV, mL	60.2 ± 13.8	87.5 ± 27.7	77.8 ± 27.4
RV EF, %	57.4 ± 5.9	52.8 ± 5.2	47.6 ± 5.8
LESV, mL	110.4 ± 13.7	140.8 ± 45.0	124.0 ± 35.7
RESV, mL	96.4 ± 16.4	135.2 ± 38.3	99.5 ± 34.7
AESV, mL	95.9 ± 15.5	148.0 ± 47.1	120.4 ± 29.3
LEF, %	21.3 ± 3.5	24.7 ± 6.6	15.0 ± 7.4
REF, %	31.4 ± 7.4	26.6 ± 8.2	33.0 ± 7.0
AEF, %	31.9 ± 3.3	20.6 ± 5.9	17.1 ± 3.4
**CMRI**			
3D RV EDV, mL	154.7 ± 23.9	200.1 ± 56.4	151.9 ± 37.6
3D RV ESV, mL	64.1 ± 10.6	92.3 ± 26.0	80.3 ± 26.6
3D RV EF, %	58.2 ± 5.7	53.6 ± 5.2	47.9 ± 5.2
LESV, mL	123.2 ± 14.9	157.4 ± 46.7	133.5 ± 38.0
RESV, mL	110.2 ± 20.3	140.3 ± 30.5	104.0 ± 33.8
AESV, mL	105.5 ± 20.0	156.0 ± 48.2	130.5 ± 33.0
LEF, %	19.9 ± 7.5	21.7 ± 4.2	12.6 ± 5.6
REF, %	28.8 ± 5.8	28.2 ± 9.8	32.5 ± 7.0
AEF, %	32.0 ± 4.2	22.6 ± 3.5	14.2 ± 2.6

*Cell values are mean ± standard deviation*.

*AEF, anteroposterior ejection fraction; CMRI, cardiac magnetic resonance imaging; EF, ejection fraction; HFrEF, heart failure with reduced left ventricular ejection fraction; LEF, longitudinal ejection fraction; REF, radial ejection fraction*.

*Other abbreviations as in Table 1*.

**Table 5:** Correlation and agreement between 3D echocardiography- and cardiac magnetic resonance imaging-derived measurements.

	**Pearson correlation**		**Bland-Altman analysis**	
	***r***	***p*-value**	**Bias**	**LOA**
RV EDV	0.971	<0.001	−11.60 mL^*^	±21.36 mL
RV ESV	0.966	<0.001	−3.74 mL^*^	±12.80 mL
RV EF	0.940	<0.001	−0.62 %	±4.57 %
LESV	0.958	<0.001	−12.94 mL^*^	±20.67 mL
RESV	0.950	<0.001	−7.82 mL^*^	±21.25 mL
AESV	0.948	<0.001	−9.23 mL^*^	±24.83 mL
LEF	0.724	<0.001	2.27 %	±10.16 %
REF	0.810	<0.001	0.52 %	±9.16 %
AEF	0.771	<0.001	0.25 %	±10.53 %

* *p < 0.05, paired Wilcoxon signed-rank test vs. null values to test the significance of the bias*.

*LOA, limits of agreement*.

*Other abbreviations as in Tables 1, 4*.

## Discussion

The detailed assessment of RV function is the cornerstone of patient management in several cardiovascular diseases. The option of measuring RV volumes and EF by 3DE opened up new horizons in terms of a relatively easy and quick but more thorough quantification of the “forgotten chamber.” 3DE-derived RV EF has a well-established added value over two-dimensional echocardiographic parameters (25). Beyond EF, however, more comprehensive metrics are needed, which can precisely characterize the complex mechanical pattern of the RV and is also able to detect subtle, often segmental dysfunction. By developing the new version of the ReVISION method, we aimed to provide a tool for the detailed evaluation of the different motion components and to enable segmental analysis using 3DE-derived RV models.

**Figure 11:**
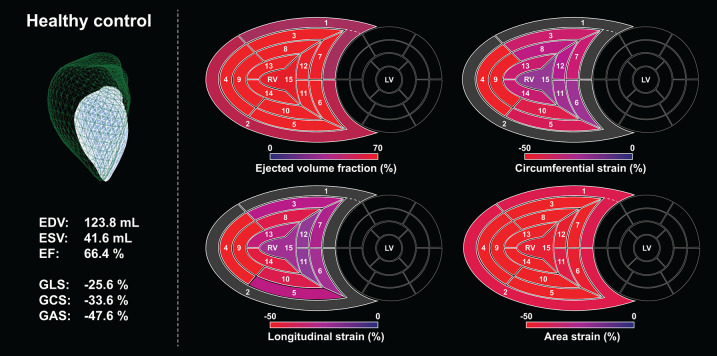
A healthy, sedentary subject's (male, 23 years old) right ventricle analyzed using the ReVISION method. Right ventricular end-diastolic volume, end-systolic volume, and ejection fraction are normal, along with global longitudinal, circumferential, and area strains. Segmental values of the aforementioned metrics at end-systole are depicted on bull's eye heatmaps. Segment numbers as defined in Figure 3. In the upper left corner, the green mesh represents the end-diastolic volume, and the blue surface corresponds to the end-systolic volume. EDV, end-diastolic volume; EF, ejection fraction; ESV, end-systolic volume; GAS, global area strain; GCS, global circumferential strain; GLS, global longitudinal strain; LV, left ventricle; RV, right ventricle.

**Figure 12:**
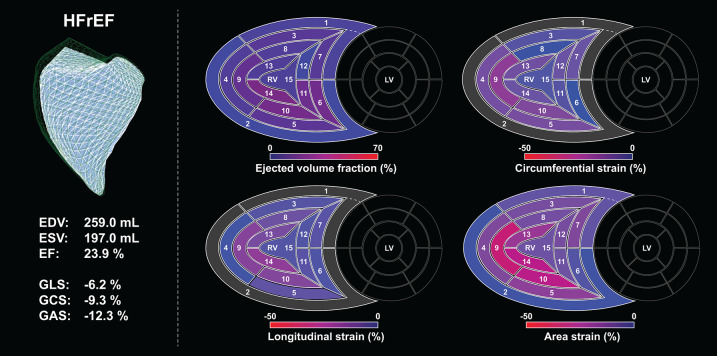
Right ventricle of a patient (male, 29 years old) with end-stage heart failure with reduced left ventricular ejection fraction analyzed using the ReVISION method. Right ventricular end-diastolic and end-systolic volumes are increased, ejection fraction is severely reduced, along with global longitudinal, circumferential, and area strains. Segmental values of the aforementioned metrics at end-systole are depicted on bull's eye heatmaps, the loss of function can be easily appreciated when compared to the maps of the healthy control (Figure 11). Segment numbers as defined in Figure 3. In the upper left corner, the green mesh represents the end-diastolic volume, and the blue surface corresponds to the end-systolic volume. EDV, end-diastolic volume; EF, ejection fraction; ESV, end-systolic volume; GAS, global area strain; GCS, global circumferential strain; GLS, global longitudinal strain; HFrEF, heart failure with reduced (left ventricular) ejection fraction; LV, left ventricle; RV, right ventricle.

In our current technical paper, we have described a novel, rule-based method for standardizing the orientation of 3D RV models, which holds the potential to progress to an agnostic approach for the analysis of 3D models provided by other vendors or imaging modalities. This is a clear step forward from our previous approach, in which each input mesh series was superimposed to a reference mesh (pre-oriented manually by expert consensus). Moreover, we have developed and tested the volumetric partitioning of the 3D model into 15 segments to allow the assessment and visualization of regional differences and to detect potential changes in the segmental contraction pattern (Figures 11, 12). The reproducibility of global and segmental volumes was found acceptable and concordant with previous reports. Finally, using a custom CMRI-based 3D reconstruction algorithm, we have found a robust agreement between 3DE- and CMRI-derived decomposed volumes and EF, confirming the applicability and credibility of our method to express the relative contribution of longitudinal, radial, and anteroposterior motion components to global RV function.

### Volumetric Segmentation of the RV

In contrast to its left counterpart, no generally accepted and standardized myocardial segmentation approach exists concerning the RV. This is attributable to its more complex 3D shape and the relatively inferior clinical relevance of segmental RV analysis in coronary artery disease compared to the LV. Nevertheless, regional alterations in RV myocardial mechanics may have a pivotal role in detecting subclinical alterations that cannot be captured by global metrics.

Various algorithms have been proposed for the volumetric segmentation of the RV. Moceri et al. used the output of the TomTec solution (4D RV-Function 2, TomTec Imaging, Unterschleissheim, Germany) and divided the 3D endocardial surface into four septal (membranous, infundibular, trabecular, inlet) and four free wall regions (inferior, lateral, anterior, outflow tract) (1). In their method, the accurate identification of regions requires the anatomical position of each vertex to be consistent across time instants and patients. In healthy subjects, they found the area and circumferential strains to show the greatest values in the inferior free wall region, whereas longitudinal deformation predominated in the inferior free wall segment. They also reported that patients with pulmonary hypertension had significantly worse longitudinal, circumferential, and area strains compared to healthy controls in all of the regions. Moreover, GAS was found to be a powerful independent predictor of survival. Similar to these results, Satriano et al. reported that principle strain (assessed with contraction angle analysis) could reliably identify pulmonary arterial hypertension patients (26). In their analysis, six surface segments (septal body, septal apex, free wall body, free wall apex, inflow, and outflow) were separated. The greatest magnitude of difference (compared to healthy controls) was observed in the principal strain values of the free wall segments, suggesting that these segments suffer the most severe contractile impairment in patients with pulmonary arterial hypertension.

Addetia and coworkers also used the triangular mesh model generated with the TomTec software solution (4D RV-Function 1.1, TomTec Imaging, Unterschleissheim, Germany) to divide the RV into four subvolumes: apex, body, inflow tract, and outflow tract (27). Then, they separated the septal and free wall surfaces of the body and the apex to analyze regional curvature indices on a total of six endocardial surfaces. In pulmonary hypertension patients, they have elegantly demonstrated that the normal “bellows effect” vanishes as the RV free wall regions remain similarly convex from end-diastole to end-systole. This finding corresponds to the literature data and our own experience with the ReVISION method, as the radial motion of the RV free wall is affected early by pressure overload, and thus, it may be a better marker of even subclinical RV dysfunction compared to global measures and, importantly, to parameters referring to longitudinal shortening only (28). To establish the reference ranges for regional indices, they quantified volumes and EFs for the four above-mentioned subvolumes and curvature for the six above-mentioned endocardial surfaces in 245 healthy subjects, and they reported sex- and age-related differences in the values of these metrics (29).

More recently, Bernardino et al. proposed an automated, mesh-independent method for partitioning the RV cavity and computing regional volumes and EFs in three regions (apex, inlet, and outflow) (30). To avoid errors due to inconsistent anatomical vertex positioning between different 3D meshes, their method uses well-defined anatomic landmarks (the apex, the tricuspid, and the pulmonary annuli), and each vertex is assigned to the region whose representing landmark is located the closest based on geodesic distance. Although they used meshes exported from the TomTec solution (4D RV-Function 2, TomTec Imaging, Unterschleissheim, Germany) in the presented analysis, their method might be capable of analyzing 3D RV models reconstructed using other post-processing software solutions as well.

Besides the methods using the triangular meshes generated with the TomTec software, there is a commercially available RV-dedicated 3D speckle tracking tool from Canon Medical Systems (Otawara, Japan) for the calculation of global and regional longitudinal, circumferential, and area strains (31). During 3D reconstruction, this software solution uses manually appointed anatomical landmarks (attachment sites of the moderator band to the septum and anterior papillary muscle to the free wall) that separate the inlet, the outflow, and the apical segments. Finally, seven surface segments are identified, namely, inlet lateral, inlet inferior, inlet septum, outflow septum, outflow free wall, apical free wall, and apical septum (32). After evaluating the reliability and feasibility of this algorithm in a group of patients with various heart diseases, Ishizu et al. suggested that the assessment of RV dyssynchrony could be one of the major clinical implications of regional RV myocardial deformation by 3D speckle tracking echocardiography (32).

Overall, clinical data support the usefulness of the 3D-derived segmental RV analysis. Although the majority of studies focused on pulmonary hypertension, we may hypothesize a similar diagnostic and prognostic value in case of RV volume overload, ischemia, or even arrhythmogenic cardiomyopathy. Of note, our software offers a unique solution for RV segmentation, as it defines 15 segments and it is capable of computing segmental EFs and longitudinal, circumferential, and area strain values.

### The Relative Contribution of Longitudinal, Radial, and Anteroposterior Motion Components to Global RV Function

In our current analysis, we found a robust agreement between 3DE- and CMRI-derived measurements of decomposed ESVs and EFs. These results also justify the concept of analyzing longitudinal, radial, and anteroposterior motions separately.

Previously, we aimed to characterize the RV mechanical pattern in healthy volunteers and various clinical settings via local and international collaborations. In a dual-center study involving 300 healthy subjects, we aimed to determine the physiological contribution of RV longitudinal, radial, and anteroposterior motion components to global RV EF (12). Despite the traditional view that the longitudinal shortening is the main driver of RV global function, we found that the anteroposterior shortening is a similarly prominent motion component. Moreover, there is an age-dependent increase (until the age of 60 years) in radial motion with a concomitant decrease in longitudinal shortening. However, individuals over 60 years of age represent a distinct group as radial motion decreases again, which may be attributable to the age-related increases in pulmonary pressures (12).

We also investigated the RV mechanical pattern of the athlete's heart. Evidently, regular vigorous physical exercise induces significant changes in cardiac morphology and function (33). The mechanical adaptation to intense, long-term exercise implies a functional shift in the RV: the relative contribution of longitudinal motion to global function was increased, whereas the radial shortening was significantly decreased (11). Moreover, this functional pattern correlated with aerobic exercise performance assessed with cardiopulmonary exercise testing, representing a potential new resting marker of the athlete's heart (11).

RV function is commonly altered following cardiac surgeries, such as coronary artery bypass grafting, surgical valve repair, or heart transplantation (34–36). The most prominent change is the decline in longitudinal shortening, even if the global RV function is preserved (10). This deterioration of the long-axis RV function is quite persistent and independent of the side of the surgical procedure (37). Notably, RV systolic function (EF and stroke volume) is mostly preserved regardless of the surgery, as radial RV contraction might compensate for the decline in longitudinal shortening. We have confirmed this phenomenon in heart transplanted patients (10). Moreover, in one of our clinical outcome studies focusing on patients with severe mitral regurgitation (PREPARE-MVR: PRediction of Early Post-operAtive Right vEntricular failure in Mitral Valve Replacement/Repair patients), the characteristic contraction pattern (i.e., decreased radial and increased longitudinal contribution to global RV function) of this patient population underwent an instantaneous shift at open-heart surgery (mitral valve replacement or repair), and the radial motion became the dominant component in the early post-operative period (13). However, at 6-month follow-up, the normal RV contraction pattern (i.e., the equal contribution of longitudinal and radial components) was restored, suggesting functional RV reverse remodeling. Interestingly, the observed increase in preoperative longitudinal contractions was associated with decreased post-operative RV contractility as assessed by right heart catheterization; thus, it might predict perioperative RV failure (13).

All these results suggest that the ReVISION software enables the exploration of RV response to different physiological and pathophysiological processes and may also provide parameters that are predictive of outcomes. Further studies are underway to explore the diagnostic and prognostic value of the 3D assessment of RV mechanics in other clinical scenarios as well.

### Limitations

The ReVISION method and the presented analyses have some limitations that should be acknowledged. First, the current version of our software relies on the 3D RV meshes generated using a specific commercially available software. However, we are continuously improving our software solution, and we are moving toward an agnostic approach that enables the processing of 3D models from other vendors or other imaging modalities as well. Second, a relatively limited number of subjects were included in our retrospective analysis. Thus, additional large-scale studies are required to evaluate the reliability, reproducibility, and repeatability of our parameters in a multicentric, prospective manner using datasets acquired with ultrasound systems of various manufacturers. Third, due to the retrospective nature of our study, same-day 3DE and CMRI examinations were not available for all subjects. In spite of this, we observed moderate-to-strong correlations between 3DE- and CMRI-derived measurements. Last, volumetric segmentation and strain analysis were not applicable to the CMRI-derived 3D meshes. Therefore, we could not assess their agreement between the two modalities.

## Conclusions

Since the first version of the ReVISION method was released, our algorithm has been continuously improved, and recently, various new features, such as the volumetric segmentation of the RV or the assessment of longitudinal, circumferential, and area strain have been implemented to enable more advanced and comprehensive analysis of the RV function using 3DE datasets. Beyond providing a detailed description of the updated ReVISION analysis pipeline, we also demonstrated the reproducibility of global and segmental RV volumes, and we compared the 3DE- and CMRI-derived metrics of the decomposed RV motion. Moreover, we updated the online interface of the software (a demo version is available at https://www.revisionmethod.com), which now allows the users to upload and analyze 3D RV meshes, to inspect the segments in 3D, to generate time–volume or time–strain curves, and to archive the findings of clinical cases. In conclusion, the ReVISION method may provide novel insights into global and also segmental RV function by defining parameters that are potentially more sensitive and predictive compared to conventional echocardiographic measurements in the context of different cardiac diseases.

## Data Availability Statement

The raw data supporting the conclusions of this article will be made available by the authors, without undue reservation.

## Ethics Statement

The study protocol conforms with the principles outlined in the Declaration of Helsinki and the local regulatory and data protection standards. All subjects in the analyzed database were enrolled as part of prospective studies (each approved by the Regional and Institutional Committee of Science and Research Ethics, approval no. 13687-0/2011-EKU and 034309-006/2014/OTIG) and provided written informed consent prior to enrollment to the archiving and analysis of their datasets and the publication of subsequent results.

## Author Contributions

MT contributed significantly to the conceptualization of the ReVISION method, participated in the software development, analyzed the data, interpreted the results, and was a major contributor to writing the manuscript. LSt and MC were major developers of the ReVISION method and helped in writing the technical section of the paper. ÁB developed the cardiac magnetic resonance imaging-based mesh reconstruction algorithm and was a major contributor to writing the technical section of the paper. BL performed the 3D echocardiographic examinations and analyzed the 3D echocardiographic datasets. FS and LSz participated in the acquisition and post-processing of the cardiac magnetic resonance imaging datasets. AF participated in the analysis of 3D echocardiographic datasets and helped in reviewing the literature. HV supervised the acquisition and post-processing of cardiac magnetic resonance imaging datasets. ZT made a major contribution to the conceptualization of the ReVISION method, supervised the software development, and participated in the interpretation of the results and manuscript preparation. BM provided the institutional background for the research. AK made a major contribution to the conception of the ReVISION method, performed the 3D echocardiographic examinations, analyzed 3D echocardiographic datasets, supervised the software development, participated in the interpretation of the results, and drafted the manuscript. All authors read and approved the final version of the manuscript.

## Conflict of Interest

ZT is a cofounder and CEO of Argus Cognitive, Inc. (the company developing the ReVISION software), holds equity in the company, and receives financial compensation for his work. LSt and MC are employees of Argus Cognitive and receive compensation for their work. The remaining authors declare that the research was conducted in the absence of any commercial or financial relationships that could be construed as a potential conflict of interest.

## Supplementary Material

Supplementary Figure 1 Correlation and agreement between right ventricular end-diastolic volume, end-systolic volume, and ejection fraction assessed using the reconstructed cardiac magnetic resonance imaging-derived meshes and those computed using the dedicated cardiac magnetic resonance imaging post-processing software. Statistical tests: the correlations between the reconstructed mesh-based measurements and the values computed using the dedicated cardiac magnetic resonance imaging post-processing software were quantified using Pearson correlation coefficients, and Bland-Altman analyses were performed to assess the bias and limits of agreement. Paired Wilcoxon signed-rank test vs. null values was applied to test the significance of the bias. 3DE, 3D echocardiography; CMRI, cardiac magnetic resonance imaging; EDV, end-diastolic volume; EF, ejection fraction; ESV, end-systolic volume; LOA, limits of agreement.
