# Supplementary figures and images for: Partitioning the Right Ventricle Into 15 Segments and Decomposing Its Motion Using 3D Echocardiography-Based Models: The Updated ReVISION Method
Source: Front Cardiovasc Med. 2021 Mar 4;8:622118. doi: 10.3389/fcvm.2021.622118 (PMC7982839; doi:10.3389/fcvm.2021.622118)

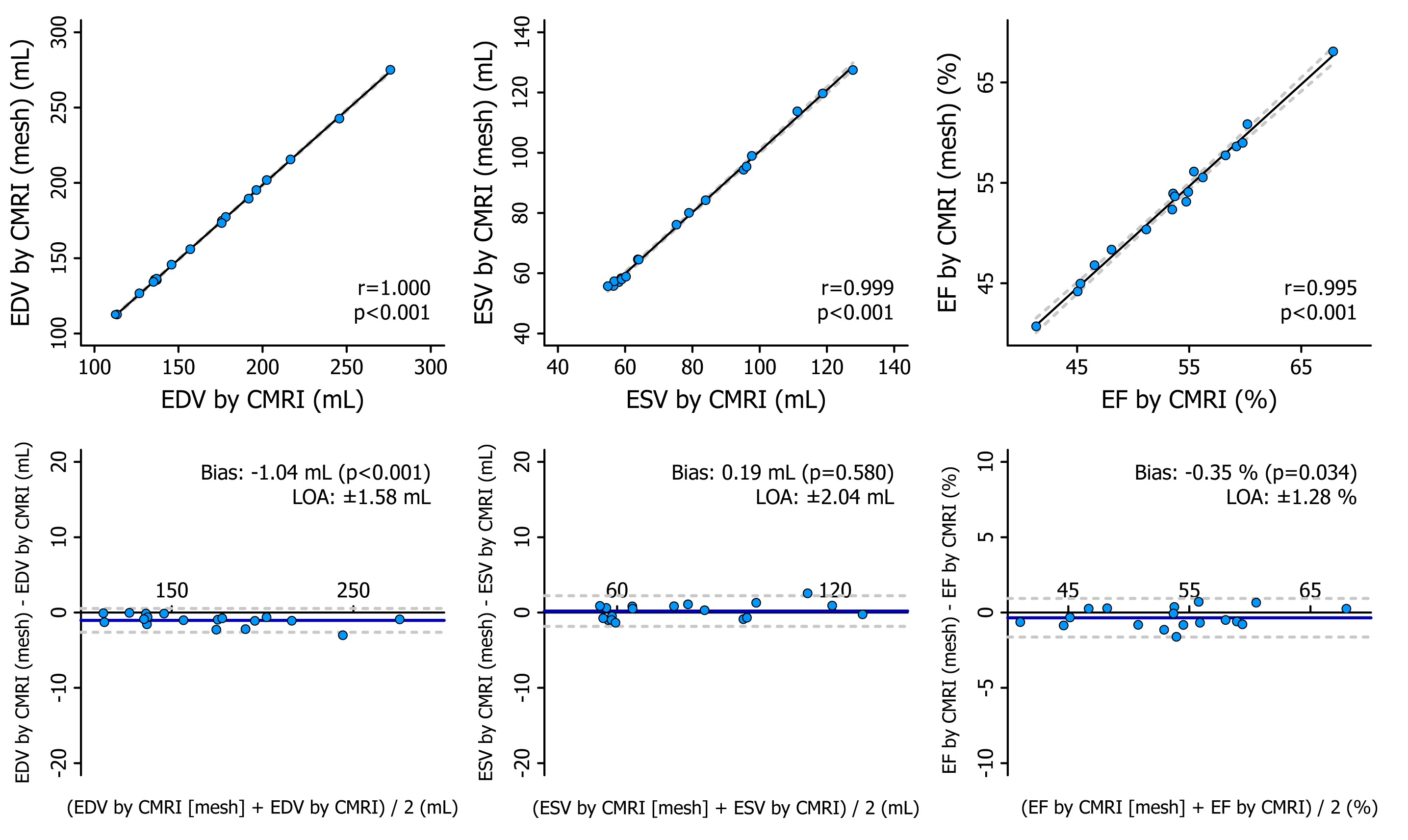

Supplement: Supplementary Figure 1 — Correlation and agreement between right ventricular end-diastolic volume, end-systolic volume, and ejection fraction assessed using the reconstructed cardiac magnetic resonance imaging-derived meshes and those computed using the dedicated cardiac magnetic resonance imaging post-processing software. Statistical tests: the correlations between the reconstructed mesh-based measurements and the values computed using the dedicated cardiac magnetic resonance imaging post-processing software were quantified using Pearson correlation coefficients, and Bland-Altman analyses were performed to assess the bias and limits of agreement. Paired Wilcoxon signed-rank test vs. null values was applied to test the significance of the bias. 3DE, 3D echocardiography; CMRI, cardiac magnetic resonance imaging; EDV, end-diastolic volume; EF, ejection fraction; ESV, end-systolic volume; LOA, limits of agreement. [file Image_1.TIF]
